# Down-Regulation of TLR and JAK/STAT Pathway Genes Is Associated with Diffuse Cutaneous Leishmaniasis: A Gene Expression Analysis in NK Cells from Patients Infected with *Leishmania mexicana*

**DOI:** 10.1371/journal.pntd.0004570

**Published:** 2016-03-31

**Authors:** Edith A. Fernández-Figueroa, Iván Imaz-Rosshandler, Juan E. Castillo-Fernández, Haydee Miranda-Ortíz, Juan C. Fernández-López, Ingeborg Becker, Claudia Rangel-Escareño

**Affiliations:** 1 Computational Genomics, Instituto Nacional de Medicina Genómica, Arenal Tepepan, México D.F., México; 2 Unidad de Investigación en Medicina Experimental, Centro de Medicina Tropical, Facultad de Medicina, Universidad Nacional Autónoma de México, Avenida, D.F., México; Charité University Medicine Berlin, GERMANY

## Abstract

An important NK-cell inhibition with reduced TNF-α, IFN-γ and TLR2 expression had previously been identified in patients with diffuse cutaneous leishmaniasis (DCL) infected with *Leishmania mexicana*. In an attempt to pinpoint alterations in the signaling pathways responsible for the NK-cell dysfunction in patients with DCL, this study aimed at identifying differences in the NK-cell response towards *Leishmania mexicana* lipophosphoglycan (LPG) between patients with localized and diffuse cutaneous leishmaniasis through gene expression profiling. Our results indicate that important genes involved in the innate immune response to *Leishmania* are down-regulated in NK cells from DCL patients, particularly TLR and JAK/STAT signaling pathways. This down-regulation showed to be independent of LPG stimulation. The study sheds new light for understanding the mechanisms that undermine the correct effector functions of NK cells in patients with diffuse cutaneous leishmaniasis contributing to a better understanding of the pathobiology of leishmaniasis.

## Author Summary

Leishmaniasis, caused by protozoan parasites is considered a neglected disease. *Leishmania mexicana* can cause localized or diffuse cutaneous leishmaniasis. Patients with localized cutaneous leishmaniasis contain the parasite within granulomas, whereas patients with diffuse cutaneous leishmaniasis show uncontrolled parasite spread. The cause of this progression remains unknown. However, NK cells have been shown to play an important role since they are among the first to produce cytokines (IFN-γ and TNF-α) that help phagocytic cells to eliminate the intracellular parasite. Previous studies had shown that NK cells of patients with diffuse cutaneous leishmaniasis are unresponsive to *Leishmania*, yet underlying mechanisms were unknown. The current work aims at understanding how the parasite modulates NK-cell responses through gene expression profiling between patients with localized and diffuse cutaneous leishmaniasis. A highlight of our results is that NK cells of patients with the uncontrolled form of leishmaniasis show down-regulation patterns for genes that regulate the innate immune response through TLR receptors and JAK/STAT signaling pathways at different levels: transcription factors (NF-κB and STAT-1), cytokine receptors (IFN-γR2 and IL-12Rβ2) and cytokines (TNF-α). The alteration of expression levels for genes in immune response signaling pathways could predispose to DCL development and/or be associated with disease severity.

## Introduction

*Leishmania mexicana* can cause two forms of clinical diseases: a benign localized cutaneous leishmaniasis (LCL) characterized by ulcers at sites of parasite inoculation, and the highly destructive and invasive form, diffuse cutaneous leishmaniasis (DCL) characterized by intensely parasitized macrophages within nodules that spread uncontrolledly throughout the skin. This aggressive form also invades the oropharyngeal and nasal mucosae of patients in an advanced stage of the disease. Although the cause of disease progression in DCL patients remains unknown, early immune events during disease development may establish conditions that determine the outcome of the infection. A cell type capable of initial immunomodulation is the NK cell since it is an early producer of IFN-γ and TNF-α, two cytokines needed to potentiate the leishmanicidal activities of phagocytic cells. It is also known that NK cells are among the first cells to produce the protective cytokines that enable macrophages to cope with the intracellular pathogen. Previous work in our lab showed that NK cells are activated by *Leishmania* lipophosphoglycan (LPG) through TLR2 receptors inducing IFN-γ and TNF-α production [1]. Another approach considered by our group was to analyze whether the cause of disease progression in DCL patients was related to an altered NK-cell response.

On activation through TLRs, NK cells initiate a signaling cascade that includes adaptor proteins such as MyD88 or TRIF. These in turn produce the recruitment and association of IRAK-1/IRAK4/TRAF-6 complex, leading to nuclear translocation of transcription factors such as IRF-3 and NF-κB as well as the production of inflammatory cytokines (IL-6, TNF-α, IL-1 and IFNs) [2,3]. NK cells can also be activated by cytokines such as IL-2, IFN-α/β, IL-12, IL-18, IL-4, TNF-α and IL-1β, alone or in a synergistic combination by binding to different receptors and activating signaling pathways such as JAK/STAT in the case of IFN-γ [4,5].

Results from our study show that DCL patients not only suffered from reduced numbers of NK cells in blood and tissue lesions, but also, that their functional capacity was markedly diminished showing a reduced production of IFN-γ and TNF-α when stimulated with *Leishmania* LPG [6]. These data strongly indicated that NK cells play a role in disease resolution of LCL patients. It became important then, to comparatively analyze genes related to the innate immune response of NK cells upon stimulation with *Leishmania* LPG in both, LCL and DCL patients. The aim of this study was to compare, at a molecular level, the response of NK cells from patients with LCL and DCL upon stimulation with *Leishmania mexicana* LPG and, to analyze whether those differences could explain the cause of disease susceptibility and/or severity in DCL patients. It was found that important genes related to immune protection against leishmaniasis, particularly those involved in the TLR and JAK/STAT signaling pathways, were down-regulated in NK cells from DCL patients. We propose that this down-regulation is possibly implicated in the susceptibility of DCL patients to *Leishmania mexicana* infections.

## Materials and Methods

### Ethical statement

This study was conducted according to the principles expressed in the Declaration of Helsinki. The study was approved by the Institutional Ethics Committee of the Medical Faculty of the National Autonomous University of Mexico (FMED/CI/RGG/013/01/2008). Guidelines established by the Mexican Health Authorities were strictly followed. All patients and healthy controls signed a written informed consent for the collection of samples and subsequent analysis.

### Patients and controls

All patients were clinically diagnosed as either LCL or DCL. This was later corroborated with laboratory tests including Giemsa-stained smears and/or immunohistochemistry of tissue lesions tested for *Leishmania mexicana*. Montenegro skin hypersensitivity tests were made at the sanitary jurisdiction office of the Cunduacan Municipality in Tabasco State, located in Southeastern Mexico, before patients began their treatment. The diagnosis was confirmed by sandwich ELISA test using total *Leishmania mexicana* antigen. All patients were from “La Chontalpa”, a region in the state of Tabasco in Mexico, which is endemic for leishmaniasis. Control samples for microarrays were obtained from donors with no history of the disease and who tested negative in the ELISA test for *Leishmania*. An ancestry analysis was included that used an additionally group of controls, these were individuals of the same geographic region that had either tested positive or negative in the ELISA test for *Leishmania*, but showed no evidence of disease. All blood samples of LCL patients were taken before they began their first treatment with Glucantime (20 mg/kg/day). DCL patients had taken their last treatment at least 3 months prior to the date that blood samples were taken for this study.

### DNA isolation

Genomic DNA was isolated from PBMC. Cells were suspended in 1 mL TRIZOL (Invitrogen Carlsbad, CA, USA), mixed and incubated for 5 min at room temperature (RT), after which 200 μL cold chloroform (Sigma) were added. The solution was mixed and centrifuged for 15 min at 4°C and 19,357 *x* g. The aqueous phase was eliminated, and the interphase and the organic phase were washed with 0.1 M sodium citrate/10% ethanol solution for 30 min under continuous mixing. The solution was centrifuged at 2,151 *x* g for 5 min at 4°C, the supernatant was discarded and the pellet was washed twice with sodium citrate, as described. One mL ethanol 75% (Sigma) was added, the solution was mixed during 10 s and centrifuged at 2,151 *x* g for 5 min at 4°C. The pellet was dried at RT, suspended in RNase free water and incubated for 15 min at 60°C. The DNA concentration was assessed using ND-1000 Spectrophotometer (NanoDrop Technologies, Wilmington, DE, USA). DNA integrity was analyzed on 1% agarose gel.

### Ancestry analysis

All patients were identified as Mexican-mestizo through a Principal Component Analysis (S1 Fig) using autosomal genome-wide data (299,411 SNPs), genotyped with Affymetrix Genome-Wide Human SNP Array 6.0 and analyzed with EIGENSTRAT Software [7, 8]. Three principal ancestral references were used, two from the HapMap International project [9] European (56 samples) and African (53 samples), and Native Mexican (71 samples) from the Mexican Genome Diversity Project (MGDP) [10,11]. The MGDP includes 21 Zapotecas from Oaxaca, 27 Mayas from Campeche and 23 Tepehuanes from Durango. The ancestry individual proportions were estimated with ADMIXTURE, V1.23 [12].

### Lipophosphoglycan (LPG) purification

Promastigotes of *Leishmania mexicana* were grown in RPMI-1640 medium (Life Technologies Laboratories, Gaithersburg, MA, USA) supplemented with 5% heat-inactivated FBS (Fetal Bovine Serum) at 28°C. For LPG extraction, promastigotes were harvested from stationary-phase cultures. Parasites were sub-cultured every 4–5 days and grown to a density of 1x10^6^/mL, centrifuged at 350 *x* g for 10 min, washed three times with cold PBS, and counted after immobilization with 0.1% glutaraldehyde. LPG was extracted from 10^10^ promastigotes, as described by McConville *et al*. [13], with some modifications. Briefly, the supernatant was removed and the pellet was extracted with chloroform/methanol/water (1:2:0.5, v/v) for 2 h at RT. The insoluble material was used for LPG extraction with 9% 1-butanol in water (2 x 500 μL) and the pooled supernatants were vacuum dried. LPG was purified from this fraction by octylsepharose chromatography in HPLC, using a 1-propanol gradient (5−60%) in 0.1 M ammonium acetate. To optimize LPG purity, two octylsepharose columns were used instead of one. The preparations tested negative for endotoxin using the *Limulus sp*. amebocyte lysate assay (E- Toxate Kit; Sigma, St. Louis, MO, USA). Polymyxin B (5 μg/mL) was also used to confirm the absence of contaminating LPS. A sample was analyzed for protein contaminants by SDS-PAGE with silver staining. The preparation was devoid of protein contaminants. Quantification of LPG was made by the Anthrone method [14].

### NK cell purification and stimulation

Peripheral blood samples of all patients and healthy volunteers was taken and kept at 4°C during 18 h before purification. Human NK cells were purified using Ficoll-Hypaque (Sigma) density gradient centrifugation at 300 *x* g for 20 min at 20°C. Cells were suspended in pyrogen-free and sterile RPMI-1640 medium (Life Technologies), supplemented with 10% heat-inactivated fetal bovine serum (FBS), 2 mM L-glutamine, 10 mM HEPES buffer, 100 μg/mL penicillin, 160 μg/mL gentamicin and 17 mM NaHCO3. PBMC were adhered for 18 h and non-adherent cells were removed, washed in PBS and thereafter purified with an NK-cell isolation Kit II, followed by magnetic sorting with MACS Microbeads (NK cell isolation kit by immunomagnetic cell sorting, Miltenyi Biotec; Bergisch Gladbach, Germany). NK cells were washed and plated in 24-well culture-plates for 18 h before assays. The purity of the enriched NK cells was assessed by flow cytometry using anti-CD56-PE and anti-CD3-FITC (Coulter Immunotech) antibodies, achieving 97% purity. NK cells were defined as CD3^-^ CD56^+^. NK cells were stimulated during 6 h with *Leishmania mexicana* LPG (20 μg/mL) at 37°C and 5% CO_2_. (The stimulation time of 6 h was based on data reviewed in the literature [15–18]). Stimulated and non-stimulated NK cells were washed twice with PBS and centrifuged at 453 *x* g for 10 min at 4°C.

### RNA isolation and microarray assays

Approximately 1x10^6^ stimulated and non-stimulated NK cells were suspended in 0.5 mL TRIZOL Reagent (Invitrogen Carlsbad, CA, USA), mixed and incubated for 5 min at RT, after which 100 μL cold chloroform were added (Sigma). This solution was mixed and centrifuged at 19,357 *x* g for 15 min at 4°C. The aqueous phase was recovered and 250 μL of cold isopropanol were added (Sigma). The resulting solution was mixed for 15 s and incubated overnight at -20°C. Thereafter, the solution was centrifuged at 19,357 *x* g for 10 min at 4°C. The supernatant was discarded and 500 μL ethanol 75% (Sigma) were added, the solution was mixed during 10 s and centrifuged at 19,357 *x* g for 10 min at 4°C. The pellet was washed with 500 μL cold absolute ethanol and centrifuged at 19,357 *x* g for 10 min at 4°C. The ethanol was discarded, the excess was air-dried at RT and the pellet was suspended in RNase free water. The RNA concentration was assessed using ND-1000 Spectrophotometer (NanoDrop Technologies, Wilmington, DE, USA). The quality of each RNA sample was validated on an Agilent BioAnalyzer 2100 (Agilent, Germany) and 150 ng of the best quality samples were processed using Affymetrix Whole Transcript Sense Target Labeling Kit (Affymetrix, Santa Clara, USA). Fragmented and labeled cDNA was hybridized onto Human Gene 1.0 ST array (Affymetrix). The arrays were washed, stained for biotinylated cDNA and scanned according to the manufacturer’s recommendations.

### Gene expression profiling

Samples were classified into three main groups (all males): (1) healthy controls, that were negative in anti-*Leishmania* ELISA test (n = 4), (2) samples from patients with LCL (n = 2), and (3) samples from patients with DCL (n = 3). Approximately 8,500 NK cells were isolated from all the samples. NK-cell mRNA of non-stimulated and LPG-stimulated groups was profiled, using Affymetrix Human Gene ST 1.0 oligo microarrays. All possible pairwise comparisons between the three groups were analyzed creating nine contrasts of interest. Three contrasts aimed at identifying genes responding to LPG stimulus: LCL_LPG *versus* LCL_NS, LCD_LPG *versus* LCD_NS, and Ctrl_LPG *versus* Ctrl_NS (NS = non-stimulated). Three more interrogate changes in expression between the non-stimulated groups and the other three between LPG-stimulated groups.

Raw data were background-corrected using Robust Multiarray Average (RMA) [19] and normalized using Quantile Normalization [20]. Differential expression was determined using statistical linear models with arbitrary coefficients, contrasts of interest were analyzed using the bioconductor library limma [21, 22]. Correction for multiple hypotheses was applied using false discovery rate (FDR) [23]. Genes were selected based on a Fold-change > 2 (Fold-Ch) and a p-value ≤ 0.05. The complete microarray analysis pipeline was conducted using Abraxas Biosystems Gene Expression Microarray Analysis Suite.

### Real-time PCR

Samples used for microarray analysis were also used for qRT-PCR validation except for the LCL group that used 4 additional samples. Total RNA (20 ng) from NK cells of 4 healthy controls, 6 LCL and 3 DCL patients (all males) were retro-transcribed using High-Capacity cDNA Archive kit (Applied Biosystems), according to manufacturer’s instructions. Relative quantification using Taqman PCR analysis was performed with the ABI PRISM 7900HT Sequence Detection System (Applied Biosystem) in a reaction volume of 20 μL containing 1X Taqman Universal Master Mix (Applied Biosystems), 1X probes and the sets of primers: Hs00765730-m1 (NF-κB1), Hs00174517-m1 (NF-κB2), Hs00936103 (IRAK-3), Hs00610101-m1 (TLR2), Hs00174128-m1 (TNF), Hs00989291-m1 (IFN-γ), Hs01013989-m1 (STAT-1), Hs00194264 (IFN-γR2), Hs01548202 (IL-12Rβ-2) and Hs01113602 (TNFAIP6) (all were Taqman Gene Expression assays, Applied Biosystems). The thermal profile was as follows: 95°C during 10 min and 40 cycles at 95°C for 15 s and 60°C for 1 min. All amplification reactions were done in duplicate and the relative quantification of gene expression was calculated using the comparative Ct method (ΔΔCt) [24]. Levels of mRNA expression were reported after normalization using GAPDH as endogenous control. GAPDH was chosen after verification that efficiencies of targets and reference gene had negligible differences. Statistically significant changes between groups were assessed using the Mann-Whitney U test. Data presented as mean +/- SEM, p< 0.05 were considered statistically significant. The analysis was done using the Prism 5 software (GraphPad Software, San Diego, CA, USA).

### Accession numbers

**TLR2:** Gene ID: 7097; UniProtKB: O60603. **IRAK3:** Gene ID: 11213; UniProtKB: Q9Y616. **NF-κB p50:** Gene ID: 4790; UniProtKB: P19838. **NF-κB p52:** Gene ID: 4791; UniProtKB: Q00653. **TNF-α:** Gene ID: 7124; UniProtKB: Q9UBM5. **IFN-γ:** Gene ID: 3458; UniProtKB: P01579. **IFN-γR2:** Gene ID: 3460; UniProtKB: P38484. **IL-12Rβ2:** Gene ID: 3595; UniProtKB: Q99665. **STAT1:** Gene ID: 6272; UniProtKB: P42224. **TNFAIP6:** Gene ID: 7130; UniProtKB: P98066.

## Results

**Fig 1:**
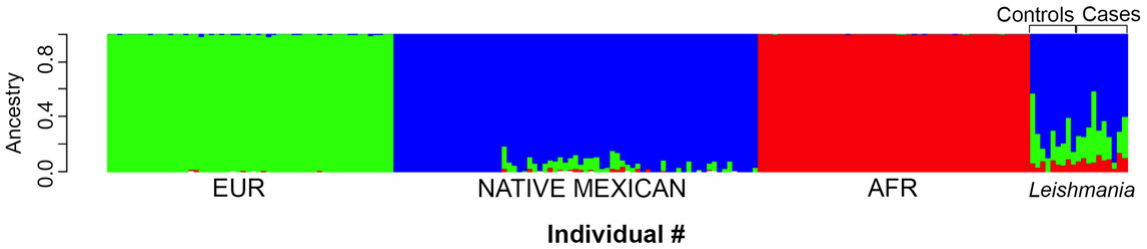
Population structure analysis. **Genome wide SNPs (299,411) were used to calculate the individual ancestry proportion of the *“Leishmania”* group, assuming the three principal ancestral populations (K = 3).** Green bars correspond to EUR (individuals with European ancestry), blue bars correspond to individuals with Native Mexican ancestry and red bars labeled as AFR correspond to individuals with African ancestry. The last block on the far right labeled “*Leishmania”* corresponds to the 9 controls and 10 cases used in this study. The X axis represents individual references for each population and the Y axis represents the ancestry proportion for each individual in a scale from 0 to 1 (colors included in each bar). The block labeled as *Leishmania* shows that all samples are essentially Mexican-mestizo.

Ancestry proportion estimates and stratification analysis were performed to rule out any bias towards genetic markers that could have an influence on the severity of the disease. The ancestry average for control samples (defined as samples of healthy individuals with positive or negative ELISA tests) was 74% Native Mexican, 21% European and 5% African. The patients (LCL and DCL) were 69% Native Mexican, 23% European and 8% African (Fig 1). In order to identify any specific ancestry differences between cases and controls along continuous axes of variation, an analysis of principal components (PCA) was performed. Results show that all the samples lie along the diagonal between the two main clusters: Caucasian and Native Mexicans, indicating some degree of mixture. Most individuals (cases and controls) were closer to the Native Mexican cluster, according to the geographic recruiting area and the origin declared by the participants in this study (S1 Fig).

### Differential expression analysis

**Fig 2:**
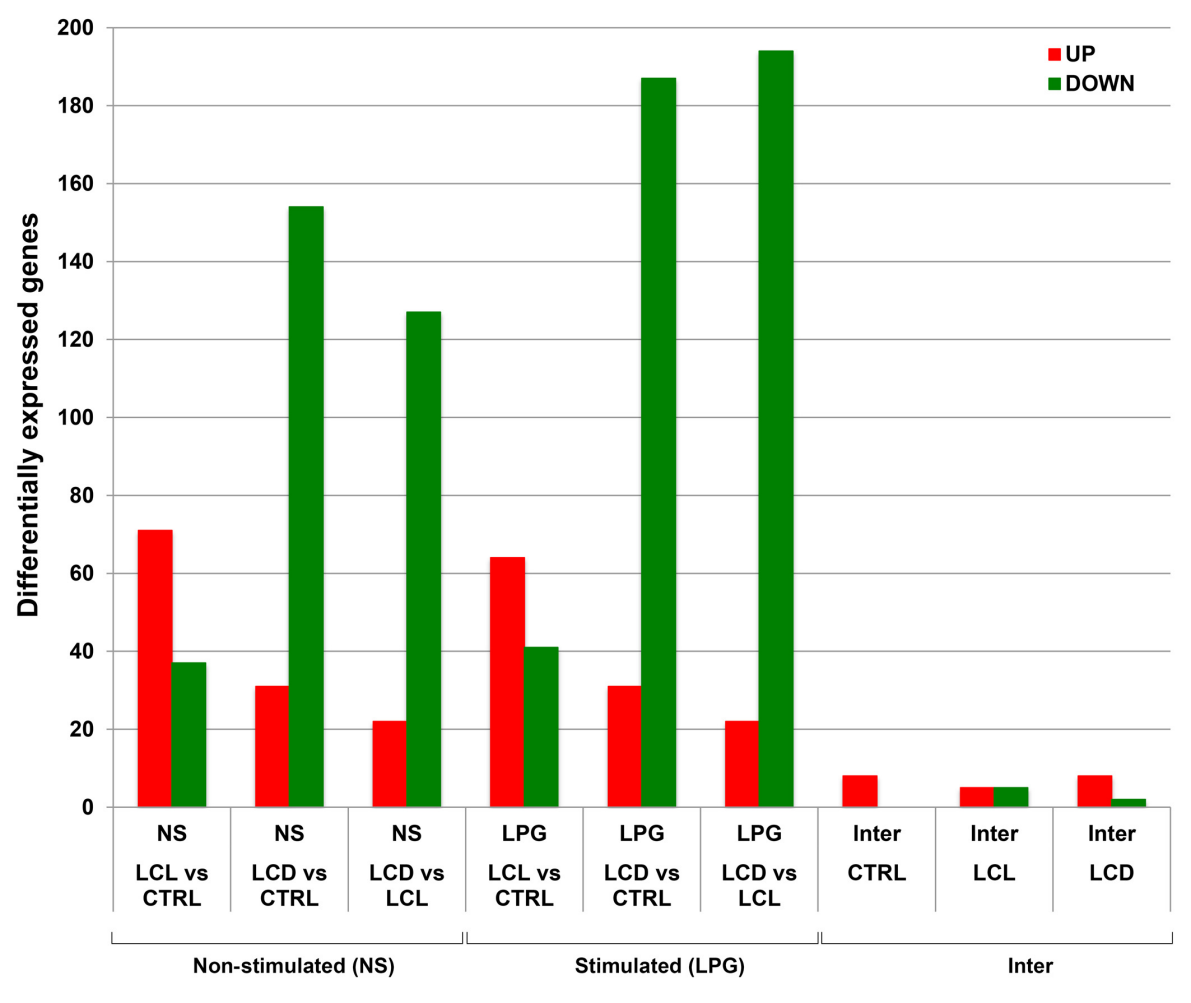
Differentially expressed genes on all nine contrasts. Bars in green represent down-regulated genes and in red those up-regulated. The Y axis represents the number of differentially expressed genes. The last six bars on the far right (Inter) show the effect of LPG stimulation (6 h) on NK cells of controls, LCL and DCL patients, revealing an almost negligible effect in all three groups. Non-stimulated NK cells (NS), LPG-stimulated NK cells (LPG) and comparison between LPG *vs* NS (Inter). Healthy controls (C), localized cutaneous leishmaniasis patients (LCL), diffuse cutaneous leishmaniasis patients (DCL).

The structure of our study, presents nine pairwise comparisons between the three groups. The numbers of differentially expressed genes per contrast are shown in Fig 2.

### The effect of LPG on gene expression

It has been reported that *L*. *major* LPG induces NF-κB nuclear translocation as well as TNF-α and IFN-γ cytokine production in healthy controls [1]. In order to verify if this phenomenon was also present at gene expression level, LPG-stimulated (LPG) versus non-stimulated (NS) NK cells from: healthy controls, LCL and DCL samples were analyzed. Results show that in the Control group (C), no genes were found to be significantly down-regulated, whereas 8 genes were significantly up-regulated (p < 0.05), 7 of which, were related to the immune response (Table 1). The activation of NK cells stimulated with *Leishmania* LPG in the control group suggests that NK cells represent a first line of innate defense against the parasite, since they respond directly to a pathogen molecule.

**Table 1:** Differentially expressed genes: C_LPG *vs* C_NS.

Gene symbol	Fold-Change	p-value	Associated to
IL-1β	10.69	0.003	Immune response
IL-1A	7.53	0.006	Immune response
IL-6	6.92	0.0002	Immune response
TNF	2.96	0.033	Immune response
IL12B	2.37	0.009	Immune response
NF-κB2	2.10	0.019	Immune response
NF-κB1	2.03	0.023	Immune response
SPRR2B	2.08	0.012	Oral squamous

These results are consistent with a previous report showing that LPG-stimulated NK cells from healthy controls have an increased TNF-α production as well as nuclear translocation of NF-κB [1]. In the case of LCL patients, the analysis of LPG-stimulated *vs* non-stimulated NK cells showed that only one gene *TCEB3CL*, associated with transcription, was down-regulated (p = 0.0007, Fold-Ch = 2.05). On the other hand, the same contrast but for DCL patients showed that two genes, both associated with mitochondrial biogenesis, were up-regulated. One of these (MT1) also had multiple up-regulated isoforms (Table 2).

**Table 2:** Differentially expressed genes: DCL_LPG *vs* DCL_NS.

Gene symbol	Fold-Change	p-value
HLA-DOB	-0.02	6.19^E-06^
MT1E	3.33	8.93^E-05^
MT1F	3.17	3.09^E-05^
MT1G	2.68	2.67^E-04^
MT1X	2.43	9.54^E-05^
MT2A	2.45	1.16^E-03^

Taken together, these results suggest that stimulation of NK cells with 20 μg of LPG during 6h up-regulates some genes associated to immune response but only in healthy controls.

The other 6 contrasts: LCL *vs* C; DCL *vs* C; LCL *vs* DCL in non-stimulated as well as LPG-stimulated present a larger number of differentially expressed genes.

### Differential expression for non-stimulated groups: controls, LCL and DCL

The comparative analysis of gene expression of LCL *vs* C showed 108 differentially expressed genes: 71 up-regulated (66%) and 37 down-regulated (34%) (Fig 2, bars 1 and 2). In contrast, for DCL *vs* C, 185 genes were found to be differentially expressed but only 31 of these were up-regulated (17%), whereas the remaining 154 genes were down-regulated (83%) (Fig 2, bars 3 and 4). A similar pattern was observed when comparing the expression of genes of DCL *vs* LCL, showing 149 differentially expressed genes: only 22 genes were up-regulated (15%) and 127 were down-regulated in DCL samples (85%) (Fig 2, bars 5 and 6). This suggests that down-regulation patterns in DCL patients may play a role in disease susceptibility.

### Differential expression after LPG-stimulation in controls, LCL and DCL samples

A similar pattern of differential gene expression was evidenced in LPG-stimulated samples, although some of the genes differed from those of non-stimulated samples. For the case of LCL *vs* C, a total of 105 genes were found to be differentially expressed: 64 up-regulated (61%) and 41 down-regulated (39%) (Fig 2, bars 7 and 8). In contrast, for DCL *vs* C a total of 218 genes were found to be differentially expressed, of which only 31 were up-regulated (14%) and 187 down-regulated (86%) (Fig 2, bars 9 and 10). Similarly, for the analysis of DCL *vs* LCL, 194 out of the 216 differentially expressed genes were down-regulated (90%), (Fig 2, bars 11 and 12).

Gene set enrichment analysis per contrast using KEGG (Kyoto Encyclopedia of Genes and Genomes) showed mainly pathways associated to immune response including Toll-like receptors, JAK/STAT, MAPK signaling pathways and cytokine-cytokine receptor interaction (All lists of differentially expressed genes and Venn diagrams are in supplementary file S1 File and KEGG enrichment analysis in S1 Table).

An additional analysis of statistically significant enriched pathways (p-value ≤ 0.05) using DAVID v6.7 (The Database for Annotation, Visualization and Integrated Discovery) was performed. Again, most of the enriched pathways were those associated to the immune system. The differentially expressed genes of the different contrasts are shown in supplementary file S2 File). The contrasts include: C_LPG vs C_NS; LCL_LPG vs LCL_NS; DCL_LPG vs DCL_NS in addition to LPG-stimulated and non-stimulated: LCL vs Controls, DCL vs Controls and DCL vs LCL.

**Fig 3:**
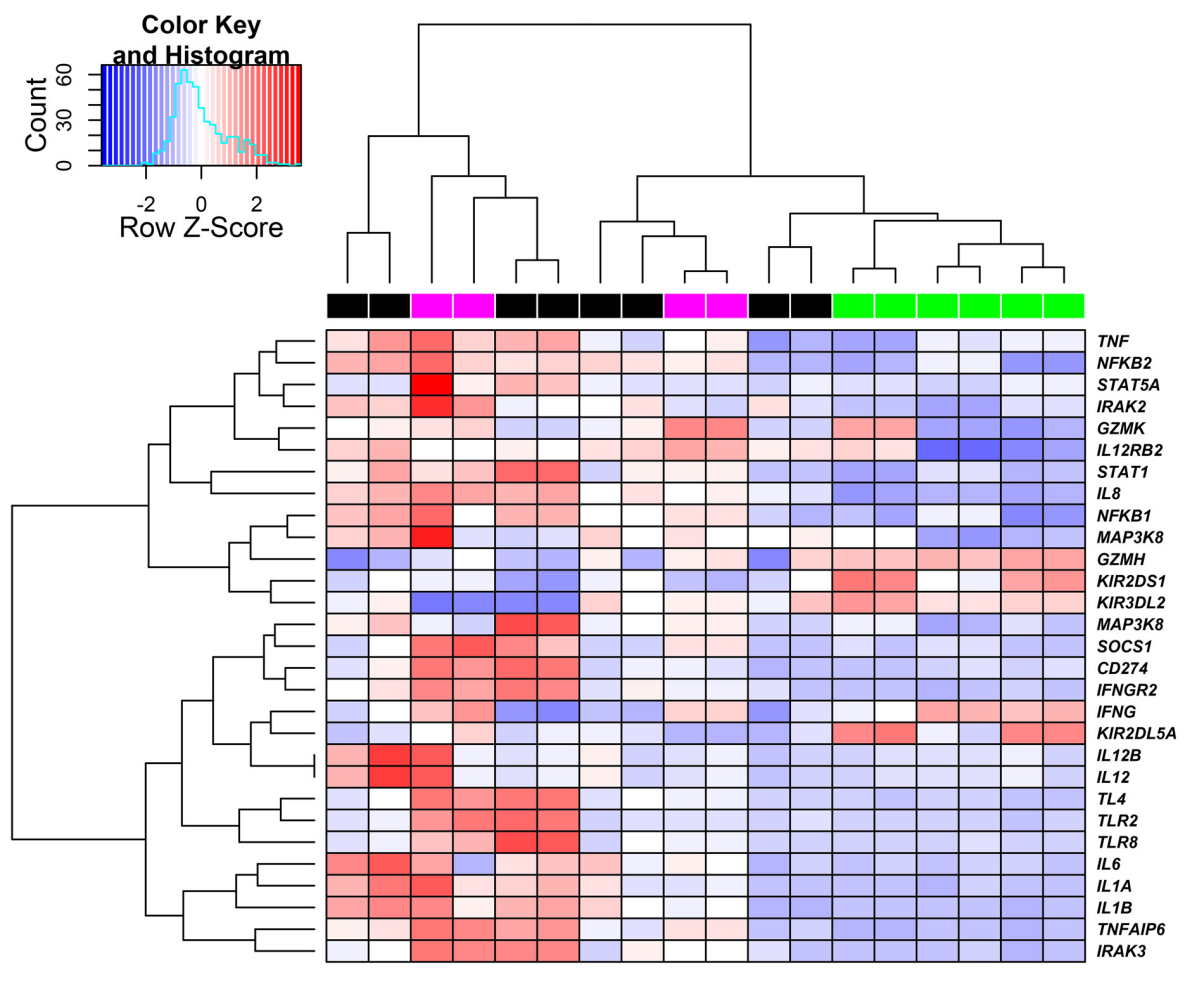
Gene expression profiles through an unsupervised hierarchical clustering approach of differentially expressed genes in both, non-stimulated and LPG-stimulated NK cells. The heatmap shows expression patterns of genes involved in the activation of the immune response. Columns represent samples, color bars on the horizontal above the heatmap label the different groups: DCL in green, LCL in magenta and Controls in black. Each row represents gene expression level across samples. Heatmap colors show up-regulation in red and down-regulated genes in blue.

To further examine these findings, genes involved in the immune response were classified using non-supervised hierarchical clustering (Fig 3).

The data show that gene expression profiles are consistent for DCL patients (labeled green top horizontal bar) but at the same time, show some opposite patterns in control (black) and LCL (magenta) samples. Note that some of the genes associated with immune response are down-regulated in DCL patients including IRAK-3, NF-κB2, IL-12Rβ-2, STAT-1 and TNF-α. It is noteworthy, that these genes have also been reported as key players in the control of *Leishmania* infections [1, 6, 25, 26]. In contrast, genes encoding IRAK-3 and TNF-α appear up-regulated in LCL patients possibly contributing in promoting a protective immune response.

### qRT-PCR validation

In order to validate the results from the microarray approach and using the same contrasts, ten genes important in the immune response against *Leishmania* were selected for relative quantification using qRT-PCR (Table 3). The list includes TLR2, IRAK-3, NFκ-B1, NF-κB2, IFN-γR2, IL-12Rβ-2, STAT-1, TNF-α and IFN-γ. Additionally, we validated TNFAIP6 because it was the gene with the largest fold change for all contrasts.

**Table 3:** Validation results for each contrast. The first column shows the microarray differential expression in Fold-change (Fold-Ch) and the second column in parenthesis shows the expression values of qRT-PCR (ΔΔCt).

				Contrast			Contrast	
				NON STIMULATED (NS)			STIMULATED (LPG)	
			LCL vs C	DCL vs C	DCL vs LCL	LCL vs C	DCL vs C	DCL vs LCL
			Fold-Ch/(ΔΔCt)	Fold-Ch/(ΔΔCt)	Fold-Ch/(ΔΔCt)	Fold-Ch/(ΔΔCt)	Fold-Ch/(ΔΔCt)	Fold-Ch/(ΔΔCt)
**Pathway**								
**TLR**		**Gene symbol**						
	**Location**	**TLR2**	*1*.*76 (3*.*65)*	**-2.05 (0.09)**	-3.63 (0.02)*	*1*.*44 (2*.*11)*	**-1.92 (0.07)**	**-2.77 (0.03)**
	**Membranal**	**IRAK-3**	*2*.*82 (2*.*69)*	-5.06 (0.07)*	-14.42* (0.02)*	*3*.*2 (2*.*17)*	**-4.89 (0.04)**	-15.77* (0.02)*
	**Intracellular**	**NF-κB1**	*1*.*21 (1*.*13)*	**-1.30 (0.80)**	-2.41* (0.70)	*1*.*20 (1*.*18)*	-1.99* (0.48)	-2.37* (0.41)
	**Nuclear**	**NF-κB2**	*1*.*62 (1*.*67)*	**-1.48 (0.67)**	-2.39* (0.39)	*1*.*15* **(0.87)**	-2.96* (0.18)*	-3.41* (0.20)*
**JAK/STAT**								
	**Membranal**	**IFN-γR2**	*1*.*22 (1*.*12)*	-2.51 (0.02)*	-3.07 (0.018)*	*1*.*25 (1*.*54)*	**-2.88 (0.18)**	-3.60* (0.20)*
	**Intracellular**	**IL-12Rβ2**	*1*.*11 (1*.*06)*	-2.14* (0.39)	-2.36* (0.37)	*1*.*10 (1*.*10)*	-2.23* (0.39)	-2.47* (0.36)
	**Nuclear**	**STAT-1**	*1*.*29 (1*.*55)*	**-1.86 (0.60)**	**-2.41 (0.39)**	**-1.29** *(1*.*08)*	-2.77* (0.39)	**-2.15 (0.36)**
**Cytokines**								
	**Secreted**	**TNF-α**	*2*.*18 (2*.*13)*	**-1.04 (0.81)**	**-2.28 (0.38)**	*1*.*20* **(0.53)**	-3.27* (0.11)	-3.91* (0.21)
		**TNFAIP6**	*9*.51* *(14*.*28)*	**-4.99 (0.10)**	-54.19* (0.007)	*3*.*73 (5*.*57)*	-15.77*(0.02)	-59.30* (0.004)*
		**IFN-γ**	*2*.71* **(0.54)**	*2*.17* **(0.86)**	**-1.24** *(2*.*79)*	*1*.99* *(1*.*32)*	*1*.96* **(0.98)**	**-1.01 (0.72)**

Non-stimulated NK cells (NS), LPG-stimulated NK cells (LPG), Healthy controls (C), localized cutaneous leishmaniasis patients (LCL), diffuse cutaneous leishmaniasis patients (DCL). Bold numbers represent down-regulated gene expressions and numbers in italics show up-regulated expressions. Values with * are p≤0.05.

Rows represent genes encoding proteins that were clustered according to the signaling pathway in which they participate as well as to their location within the cell. Columns represent our main six contrasts each arranged in two sub-columns one for differential expression in fold change and the second for qRT-PCR differential values in ΔΔCt. Two blocks represent non-stimulated, and LPG-stimulated on the far right. Up-regulation is marked in italics and down-regulation is marked in bold black. It is observed that the down-regulation pattern for qRT-PCR is closely similar to that for microarray data. According to results in Table 3, most values were validated through qRT-PCR except for IFN-γ that shows a persistent inconsistency. A possible explanation for this could be the reduced sample size.

## Discussion

*Leishmania mexicana* can cause two clinical forms of cutaneous leishmaniasis with contrasting severity. Patients with less severe clinical form, LCL, present ulcers at the sites of the sand fly bite that contain relatively low numbers of parasites. In contrast, patients with DCL have nodules containing highly parasitized macrophages that spread uncontrollably throughout the skin. Whilst LCL patients are able to contain the spread of the parasite, DCL patients are unable to control parasite reproduction inside macrophages, which eventually burst, releasing infective amastigotes. These are taken up by other phagocytic cells such as neutrophils, macrophages and dendritic cells, thereby protecting the parasites from the deleterious effect of complement activation. Their “Trojan horse” strategy, originally described by Wilson *et al*., permits parasites an early escape from the infection site. Once inside phagocytic cells, they can inhibit microbicidal mechanisms such as the generation of NO and reactive oxygen metabolites, both of which, are highly toxic for the parasite [27, 28]. Phagocytic cells need to be activated in order to cope with intracellular *Leishmania* infections. Cytokines such as IFN-γ and TNF-α are crucial for inducing leishmanicidal mechanisms in phagocytic cells. In addition to activating the infected cells, these two cytokines also favor the formation of granulomas in the infected tissues in an effort to contain parasite spread [29]. This could be evidenced in lesions of LCL patients, where well-organized granulomas have been reported. In contrast, lesions of DCL patients show diffusely scattered cells consisting mainly of heavily vacuolated macrophages harboring abundant amastigotes [2, 30]. Taken together, these evidences highlight the importance of IFN-γ and TNF-α for *Leishmania* control and their lack of presence in DCL patients that could ultimately be responsible for the uncontrolled parasite spread. The protective effect of IFN-γ is achieved through various mechanisms: 1) by induction of the expression of NO synthase 2 (NOS2) gene; 2) favoring a Th1 polarization of CD4^+^ T cells (thereby guaranteeing further IFN-γ production); and 3) by inducing maturation of dendritic cells and their migration to lymph nodes [6, 31, 32].

NK cells are among the first to produce IFN-γ and TNF-α in response to pathogen presence and in leishmaniasis, these cells can become activated after the binding of TLR2 to *Leishmania* LPG [1]. The possible role of NK cells in defining disease outcome in patients infected with *L*. *mexicana* was reported in previous work of our group [2]. We were able to show that effector functions in NK cells differ between LCL and DCL patients. For instance, NK cells of DCL patients showed an important reduction in IFN-γ and TNF-α production and reduced expression of TLR2, TLR1 and TLR6, as compared to NK cells of LCL patients, which showed an enhanced production of both cytokines and TLR expressions after stimulation with LPG. Moreover, we observed a reduced number of NK cells in peripheral blood and tissue lesions as well as down-regulation of IFN-γ in DCL patients by qRT-PCR. This evidence on impaired NK-cell function in DCL patients and the possible correlation to the disease severity, called for a precise analysis of the mechanisms involved. We therefore used NK cells of the same LCL and DCL patients to analyze the expression of the genes involved in innate cytokine production and TLR signaling pathways. We now show that disease severity correlates with the down-regulation of important genes involved in the early immune response of DCL patients. We were also able to validate most of the genes associated to innate immune response by qRT-PCR, finding a robust consistency with the gene expression patterns obtained from the microarray analysis, some of which were statistically significant in both platforms. The down-regulation of genes encoding IRAK-3, NF-κB2, IL-12Rβ2, STAT-1 and TNF-α in DCL patients intervenes at different levels in the pathway needed for the activation of NK cells and their IFN-γ and TNF-α production. The process begins with IL-12 production mediated by activation of the TLR2 signaling pathway, which in turn is required for NK-cell stimulation and for a CD4^+^ Th1 development [33]. Previous studies have associated IL-12 with protection against *L*. *major* infections [6, 34]. Our data now show that NK cells of DCL patients have reduced expression levels for IL-12Rβ2, hindering an early NK response to IL-12 stimulation. Furthermore, down-regulation of TLR2 in these patients also impairs binding of *Leishmania* LPG, which can affect NK-cell activation at two levels. First, it hampers nuclear translocation of NF-κB and next it interferes with JAK/STAT activation that is initiated by the crosstalk between TLR/IL-1 and JAK/STAT signaling pathways. This is in accordance with Luu *et al*., who recently described that STAT-1 interacts with TRAF6 following TLR activation and that the phosphorylation of STAT-1 has a critical role in augmenting TLR-induced NF-κB activation [4]. Our data also show that in addition to reduced TLR2 and STAT-1, the expression levels of genes encoding NF-κB1 and NF-κB2 are also down-regulated in DCL patients. This suggests that the blockage of the TLR and STAT signaling pathways may be critical in activation and regulation of the pro-inflammatory responses following pathogen challenge. The inhibition of the immune response is further augmented by the low expression of IFN-γR2 in NK cells of DCL patients. Since IFN-γ is one of the key cytokines needed for the protective immune response against *Leishmania*, and its biological functions are mediated by activation of JAK/STAT kinases [35], the down-regulation of IFN-γR2 and STAT-1 genes possibly render NK cells of DCL patients unresponsive to this cytokine.

The importance of TLR receptors in the protection against *Leishmania* has also been shown in mouse models. Results indicate that resistance to *L*. *major* infections is induced by IL-12 and associated with the MyD-88-dependent pathway on TLR activation leading to a Th1 response. This contrasts with the disease susceptibility and the Th2 response observed in MyD-88^−/−^ mice [3].

It is noteworthy that LPG stimulation led to a transient up-regulation of IFN-γ gene expression in NK cells of both groups of patients, as seen in Table 3. Yet, the biological implications of the elevated gene expression in DCL patients are not evident since these patients showed down-regulation of the receptor IFN-γR2. Hence, only LCL patients seem to be able to benefit from this cytokine. Interestingly, *in vitro* stimulation of NK cells with LPG further down-regulated the expression of the IFN-γR2 gene in DCL patients, although it did not modify the expression of STAT-1 and IL-12Rβ2 genes. With these results we are tempted to speculate that IFN-γR2 in NK cells of DCL patients is more susceptible to modulation by *L*. *mexicana* LPG, as compared to STAT-1 and IL-12Rβ2. Our evidence is in accordance with the literature, where C57BL/6 STAT-1^-/-^ mice, infected with *L*. *major*, showed a reduction of IL-12, IFN-γ and nitric oxide (NO) production, and developed larger lesions containing significantly more parasites as compared to WT C57BL/6 mice [5].

All points to the idea that strong reduction in gene expression of TLR2, IRAK-3, NF-κB1, NF-κB2, IFN-γR2, IL-12Rβ2, STAT-1 and TNF-α may block NK-cell activation and effector mechanisms in DCL patients through various mechanisms: 1) reduced expression of IL-12Rβ2 limits IL-12 stimulation; 2) down-regulation of STAT-1 gene interferes with IFN-γ production; 3) down-regulation of IFN-γR2 interferes with autocrine activation of NK cells as well as of other immune cells. Thus, several of the critical early protective molecules, receptors and mechanisms needed for protection against *Leishmania* are likely to be shut down, leaving DCL patients unprotected against parasite replication.

The cause of down-regulation of genes in DCL patients remains unknown, though one may speculate that DNA methylation or other epigenetic modulation could play a role. It has been shown that the response of immune cells to invading pathogens can lead to genomic instability and DNA damage. Furthermore, intracellular pathogens can alter the epigenome integrity of the host, possibly through DNA methylation or regulation of microRNA (miRNA) [36, 37]. MicroRNAs are post-transcriptional regulators that belong to a molecular regulation system known as RNA interference and immune responses can be regulated by pathogen-encoded miRNAs [38]. MicroRNAs have been detected in macrophages and dendritic cells infected with *L*. *donovani* and *L*. *major*, respectively, and have been proposed to be responsible for epigenetic changes in DNA methylation [39, 40]. Even though and to the best of our knowledge, *Leishmania mexicana* infections have not been related to microRNAs capable of regulating NK cells, it remains to be analyzed whether microRNAs can be associated with impaired NK effector functions and disease development, as has been demonstrated for other infectious diseases [41]. It would be interesting to establish whether these parasites possibly modify the host immune response at a molecular level through microRNAs, thereby modifying immune cell molecules and mechanisms as part of their evasion strategies. This has been shown for *L*. *donovani* that causes abnormal nuclear translocation of STAT-1 leading to: (1) its rapid proteosomal degradation, (2) diminished levels of the IFN-γ receptor α-chain and (3) the induction of SOCS3, a negative regulator of IFN-γ signaling [42].

Evidence that the parasite can attenuate IFN-γ-induced tyrosine phosphorylation, inhibition of the alpha subunit of IFN-γR expression and a transient induction of SOCS3 has been presented [25, 26]. In accordance, *L*. *mexicana* has been shown to block IFN-γ mediated NO production in infected macrophages and to increase protein-tyrosine phosphatase activity, particularly of SHP-1. Additionally, this parasite causes elimination of the p65-containing subunit of NF-κB, cleaving p65 into a p35-containing subunit and promoting total protein degradation. It also inhibits STAT-1 and AP-1 activity [43]. Moreover, *Leishmania* LPG binding to TLR2 can induce the expression and activation of the serine/threonine phosphatase PP2A that inactivates TLR cytoplasmic adaptor proteins (IRAK-1, MAPKs, and IκB), thereby leading to tolerance [31]. All, indicating that the parasite has developed complex evasion strategies that can inhibit critical effector mechanisms of the immune response, the impact of which requires to be analyzed in the human host.

Another remark is that in LCL patients, TLR2, IRAK-3 and NF-κB1 genes were up regulated, both in non-stimulated and LPG-stimulated samples. These findings could explain why NK cells in LCL patients produce IFN-γ and TNF-α and over-express membrane TLR2 when these cells are stimulated with LPG [2]. Interestingly, *Leishmania* infections in LCL patients achieved maximal up-regulation for TLR2, IRAK-3 and NF-κB1, which was not modified by further in vitro stimulation with LPG.

In summary, results of this study validate our earlier observations on the important role of NK cells in conferring an early protective response. We are able to show for the first time in samples from patients infected with *L*. *mexicana*, that important innate immune-related genes are down-regulated in DCL patients beginning at the early stages of the infection, which possibly interferes with an adequate protective response against *Leishmania*.

TNFAIP6 (TSG-6), the gene with largest fold-change was down-regulated in DCL patients (-54.19 in NS and -59.3 in LPG-stimulated NK cells), is expressed by many different cell types in response to pro-inflammatory cytokines and encodes a protein that is secreted at inflammation sites, playing an important role in the protection of tissues from the damage of acute inflammation [44]. Also, this protein interacts with CD44R on resident macrophages. The fact that TNFAIP6 (TSG-6) has not been previously reported in leishmaniasis, and due to the important level of down-regulation found in DCL patients, its role still needs to be analyzed at the functional level.

In conclusion, the down-regulation of genes that contribute in the immune response regulation of both TLR and JAK/STAT signaling pathways affect different molecules of NK cells: transcription factors (NF-κB and STAT-1), cytokine receptors (IFN-γR2 and IL-12Rβ2) and cytokines (TNF-α). These findings seem to correlate with the more severe clinical form of cutaneous leishmaniasis (DCL).

The clear pattern of a large number of down-regulated genes in DCL samples before and after being stimulated with *Leishmania* LPG suggests a possible association between gene regulation and disease susceptibility and/or severity. Thus, development of the clinical form of LCL or DCL may be associated to down-regulation gene patterns in NK cells. However, it remains to be demonstrated whether and how these down-regulated genes favor dissemination of *Leishmania mexicana* in these patients.

## Supporting Information

S1 Fig Principal Components Analysis (PCA). The two most informative eigenvectors were plotted in 9 Controls and 10 LCD and LCL cases, all of them Mestizo and HapMap populations 53 African (YRI), 56 European (CEU) and 71 Native Mexican (NAT) (21 Zapotecas, 27 Mayas and 23 Tepehuanes). The Mestizo samples in this study show short distance to the Native Mexican cluster.
(TIF)

S1 Table KEGG analysis of genes that are up-regulated or down-regulated in NK cells with a fold change > 2 and a p-value ≤ 0.5. (DOC)

S1 File Overlapping genes and Venn diagrams for non-stimulated vs LPG-stimulated samples. (XLS)

S2 File DAVID gene set enrichment analysis for up- and down-regulated genes with at least 2-fold changes and p-value ≤ 0.05. (XLS)
