# Correction: Down-Regulation of TLR and JAK/STAT Pathway Genes Is Associated with Diffuse Cutaneous Leishmaniasis: A Gene Expression Analysis in NK Cells from Patients Infected with *Leishmania Mexicana*

**DOI:** 10.1371/journal.pntd.0004666

**Published:** 2016-04-20

**Authors:** The PLOS Neglected Tropical Diseases Staff

There are errors in the author affiliations. The affiliations should appear as show here:

Edith A. Fernández-Figueroa^2^, Iván Imaz-Rosshandler^1^, Juan E. Castillo-Fernández^1^, Haydee Miranda-Ortíz^1^, Juan C. Fernández-López^1^, Ingeborg Becker^2^, Claudia Rangel-Escareño^1^

**1** Computational Genomics, Instituto Nacional de Medicina Genómica, Periférico Sur 4809, Arenal Tepepan, 14610, México D.F., México, **2** Unidad de Investigación en Medicina Experimental, Centro de Medicina Tropical, Facultad de Medicina, Universidad Nacional Autónoma de México, Avenida Universidad 3000, C.P.04510 México, D.F., México.
