# Supplementary figures and images for: Down-Regulation of TLR and JAK/STAT Pathway Genes Is Associated with Diffuse Cutaneous Leishmaniasis: A Gene Expression Analysis in NK Cells from Patients Infected with Leishmania mexicana
Source: PLoS Negl Trop Dis. 2016 Mar 31;10(3):e0004570. doi: 10.1371/journal.pntd.0004570 (PMC4816531; doi:10.1371/journal.pntd.0004570)

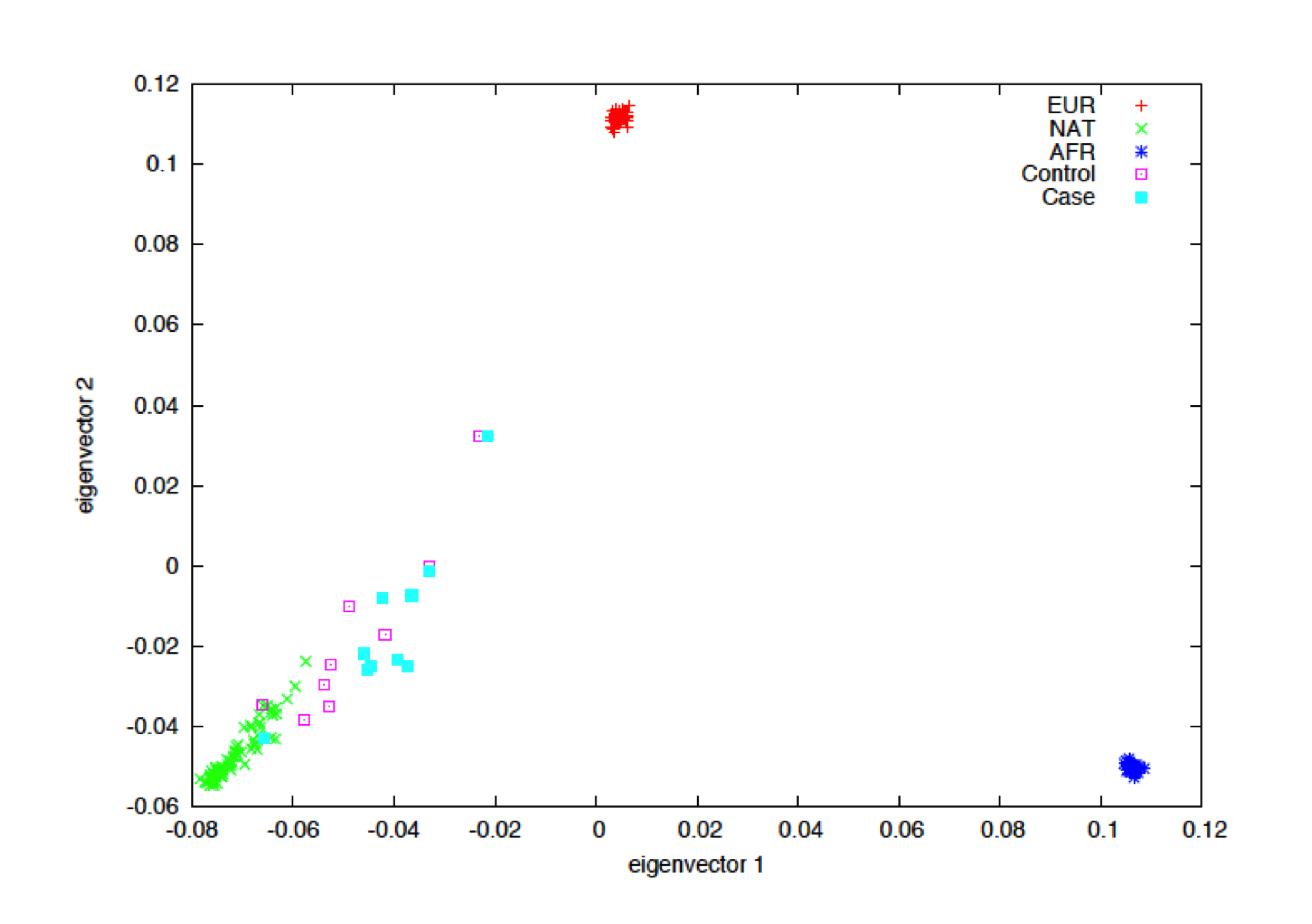

Supplement: S1 Fig — The two most informative eigenvectors were plotted in 9 Controls and 10 LCD and LCL cases, all of them Mestizo and HapMap populations 53 African (YRI), 56 European (CEU) and 71 Native Mexican (NAT) (21 Zapotecas, 27 Mayas and 23 Tepehuanes). The Mestizo samples in this study show short distance to the Native Mexican cluster. (TIF) [file pntd.0004570.s001.tif]
