# Supplementary material for: Down-Regulation of TLR and JAK/STAT Pathway Genes Is Associated with Diffuse Cutaneous Leishmaniasis: A Gene Expression Analysis in NK Cells from Patients Infected with Leishmania mexicana
Source: PLoS Negl Trop Dis. 2016 Mar 31;10(3):e0004570. doi: 10.1371/journal.pntd.0004570 (PMC4816531; doi:10.1371/journal.pntd.0004570)
Supplement: S1 Table — (DOC) [file pntd.0004570.s002.doc]

**Supplementary Material S1**

Table S1. KEGG analysis of genes up- and down-regulated in non-stimulated and LPG-stimulated NK cells with a fold change > 2 and a p-value ≤ 0.5 for each contrast. C=Controls; LCL=Patients with localized cutaneous leishmaniasis; DCL=Patients with diffuse cutaneous leishmaniasis; NS=Non-stimulated cells; LPG= LPG-stimulated cells.

| **Contrast** | **Pathway** |
| --- | --- |
| **Inter** |  |
| **C_LPG *vs* C_NS Up** | Cytokine-cytokine receptor interaction |
|  | Leishmaniasis |
|  | Toll-like receptors signaling pathway |
|  | NOD-like receptor signaling pathway |
| **LCL_LPG *vs* LCL_NS Down** |  |
|  | Olfatory transduction |
| **LCL_LPG *vs* LCL_NS Up** | Cytokine-cytokine receptor interaction |
|  | TNF signaling pathway |
|  | NOD-like receptor signaling pathway |
|  | Chemokine signaling pathway |
|  | Toll-like receptors signaling pathway |
|  | JAK/STAT signaling pathway |
| **DCL_LPG *vs* DCL_NS Down** |  |
|  | Inflammatory bowel disease |
|  | Phagosome |
|  | Cell Adhesion molecules |
|  | Leishmaniasis |
| **DCL_LPG *vs* DCL_NS Up** |  |
|  | Mineral absorption |
| **Non-stimulated (NS)** |  |
| **LCL_NS *vs* C_NS Down** |  |
|  | Cytokine-cytokine receptor interaction |
|  | Chemokine signaling pathway |
|  | Transcriptional misregulation in cancer |
|  | MicroRNAs in cancer |
|  | ECM-receptor interaction |
|  | Cell adhesion molecules |
|  | PI3K-AKT signaling pathway |
| **LCL_NS *vs* C_NS Up** |  |
|  | MAPK signaling pathway |
|  | Protein processing in endoplasmic reticulum |
|  | Osteoclast differentiation |
|  | Antigen processing and presentation |
|  | Endocytosis |
|  | Spliceosome |
|  | Cytokine-cytokine receptor interaction |
|  | NF-kB signaling pathway |
| **DCL_NS *vs* C_NS Down** |  |
|  | Cytokine-cytokine receptor interaction |
|  | Metabolic pathways |
|  | Chemokine signaling pathway |
|  | Cell adhesion molecules |
|  | JAK/STAT signaling pathway |
| **DCL_NS *vs* C_NS Up** |  |
|  | Antigen processing and presentation |
|  | Natural killer cell mediated cytotoxicity |
|  | Leishmaniasis |
| **DCL_NS *vs* LCL_NS Down** |  |
|  | MAPK signaling pathway |
|  | Osteoclast differentiation |
|  | Protein processing in endoplasmatic reticulum |
|  | Metabolic pathways |
|  | Cytokine-cytokine receptor interaction |
| **DCL_NS *vs* LCL_NS Up** |  |
|  | Cell adhesion molecules |
|  | Antigen processing and presentation |
|  | Bladder cancer |
| **LPG-stimulated (LPG)** |  |
| **LCL_LPG *vs* C_LPG Down** |  |
|  | Cytokine-cytokine receptor interaction |
|  | Chemokine signaling pathway |
|  | Transcriptional misregulation in cancer |
|  | MicroRNAs in cancer |
|  | JAK/STAT signaling pathway |
|  | Oxytocin signaling pathway |
| **LCL_LPG *vs* C_LPG Up** |  |
|  | Estrogen signaling pathway |
|  | Protein processing in endoplasmic reticulum |
|  | MAPK signaling pathway |
|  | Cytokine-cytokine receptor interaction |
|  | Spliceosome |
|  | Antigen processing and presentation |
|  | Chemokine signaling pathway |
|  | Toll-like receptors signaling pathway |
| **DCL_LPG *vs* C_LPG Down** |  |
|  | Cytokine-cytokine receptor interaction |
|  | Rheumatoid arthritis |
|  | Chemokine signaling pathway |
|  | Metabolic pathways |
|  | JAK/STAT signaling pathway |
|  | TNF signaling pathway |
|  | Leishmaniasis |
|  | MAPK signaling pathway |
|  | NF-kB signaling pathway |
|  | Toll-like receptors signaling pathway |
| **DCL_LPG *vs* C_LPG Up** |  |
|  | Bile secretion |
|  | T cell receptor signaling pathway |
|  | Mineral absorption |
|  | Cell adhesion molecules |
| **DCL_LPG *vs* LCL_LPG Down** |  |
|  | Cytokine-cytokine receptor interaction |
|  | MAPK signaling pathway |
|  | NF-kB signaling pathway |
|  | TNF signaling pathway |
|  | Osteoclast differentiation |
|  | Toll-like receptors signaling pathway |
|  | NOD-like receptor signaling pathway |
|  | PI3K-AKT signaling pathway |
|  | JAK/STAT signaling pathway |
| **DCL_LPG *vs* LCL_LPG Up** |  |
|  | Antigen processing and presentation |
|  | Graft versus host disease |
|  | Pathway cancer |
|  | Natural killer cell mediated cytotoxicity |
|  | Cell adhesion molecules |
